# *Bacillus velezensis* GH1-13 enhances drought tolerance in rice by reducing the accumulation of reactive oxygen species

**DOI:** 10.3389/fpls.2024.1432494

**Published:** 2024-09-25

**Authors:** Dongryeol Park, Jinwoo Jang, Deok Hyun Seo, Yangseon Kim, Geupil Jang

**Affiliations:** ^1^ School of Biological Sciences and Technology, Chonnam National University, Gwangju, Republic of Korea; ^2^ Department of Research and Development, Center for Industrialization of Agricultural and Livestock Microorganisms, Jeongeup-si, Republic of Korea

**Keywords:** abiotic stress, *Bacillus velezensis*, drought, jasmonic acid, reactive oxygen species, rice

## Abstract

Plant growth-promoting rhizobacteria colonize the rhizosphere through dynamic and intricate interactions with plants, thereby providing various benefits and contributing to plant growth. Moreover, increasing evidence suggests that plant growth-promoting rhizobacteria affect plant tolerance to abiotic stress, but the underlying molecular mechanisms remain largely unknown. In this study, we investigated the effect of *Bacillus velezensis* strain GH1-13 on drought stress tolerance in rice. Phenotypical analysis, including the measurement of chlorophyll content and survival rate, showed that *B. velezensis* GH1-13 enhances rice tolerance to drought stress. Additionally, visualizing ROS levels and quantifying the expression of ROS-scavenging genes revealed that GH1-13 treatment reduces ROS accumulation under drought stress by activating the expression of antioxidant genes. Furthermore, the GH1-13 treatment stimulated the jasmonic acid response, which is a key phytohormone that mediates plant stress tolerance. Together with the result that jasmonic acid treatment promotes the expression of antioxidant genes, these findings indicate that *B. velezensis* GH1-13 improves drought tolerance in rice by reducing ROS accumulation and suggest that activation of the jasmonic acid response is deeply involved in this process.

## Introduction

Drought is a major abiotic stress that impedes crop growth and productivity. Global climate change is increasing the frequency and intensity of drought stress, and interactions with other abiotic stresses such as salinity and heat stress exacerbate the harmful effect on crops (Panda et al., 2021; Ramegowda et al., 2024). Rice cultivated in paddy fields serves as a staple food for more than half of the world’s population and is highly susceptible to drought stress (Seck et al., 2012; Seo et al., 2020; Zargar et al., 2022). Recent reports indicate that drought stress can reduce rice yields by approximately 13%–75% (Bhutta et al., 2019; Zargar et al., 2022). Therefore, various strategies to improve drought tolerance in rice have been investigated, including the utilization of plant microbiomes.

Plant growth-promoting rhizobacteria (PGPRs) are microorganisms that inhabit the rhizosphere and enhance plant growth through processes such as nutrient solubilization, nitrogen fixation, and hormone production (Lugtenberg and Kamilova, 2009; Murali et al., 2021a; Gowtham et al., 2022; Sujkowska-Rybkowska et al., 2022; Al-Turki et al., 2023). *Bacillus thuringiensis* S7, *Herbaspirillum seropedicae* ZA15, and *Klebsiella oxytoca* LCK121 have been found to enhance the growth and productivity of cereal crops, such as rice and barley, by promoting phosphorus solubilization (Estrada et al., 2013; Xiao et al., 2020; Khalifa and Aldayel, 2022). *Bacillus pumilus* JM52 and *Rhizobium daejeonense* JR5 enhanced shoot and root growth in rice by increasing soil ammonium levels, and *Arthrobacter chlorophenolicus* BHU3 improved plant height and grain yield in wheat by enhancing nitrogen uptake (Habibi et al., 2014; Kumar et al., 2014). Additionally, it was reported that *Bacillus flexus* P4 and *Azospirillum brasilense* RA-17 stimulate crop growth by promoting the production of phytohormones such as auxin and cytokinin (Ahmed and Hasnain, 2010; Zaheer et al., 2022). Furthermore, increasing research suggests that PGPRs are involved in enhancing plant response and tolerance to abiotic stresses, including drought (Gowtham et al., 2020; Murali et al., 2021b). For example, *Bacillus paramycoides* DT-85 and *Bacillus licheniformis* K11 were found to enhance drought tolerance in wheat and peppers, and *Bacillus aryabhattai* B8W22 and *Bacillus halotolerans* MSR-H4 improved salt stress tolerance in rice and wheat (Lim and Kim, 2013; El-Akhdar et al., 2020; Shultana et al., 2021; Yadav et al., 2022). Moreover, it has been reported that *Achromobacter* strains, including *Achromobacter xylosoxidans* Cm4 and *Achromobacter piechaudii* ARV8, as well as *Pseudomonas* strains, including *Pseudomonas psychrotolerans* CS51 and *Pseudomonas stutzeri* C4, exhibit the ability to enhance abiotic stress tolerance in various crops such as potato, peppers, maize, and tomato (Mayak et al., 2004; Tank and Saraf, 2010; Belimov et al., 2015; Kubi et al., 2021).

Reactive oxygen species (ROS) are highly reactive due to the presence of unpaired electrons, and they are naturally produced as by-products of cellular metabolic processes, such as in the photosynthetic system in chloroplasts and the cellular respiration system in mitochondria (Dat et al., 2000; Mittler, 2002; Sharma et al., 2012). When ROS levels are excessively increased in cells, it can directly cause lipid peroxidation, protein carbonylation, and DNA damage, which leads to loss of membrane integrity, enzyme activity inhibition, and cell death (Tripathy and Oelmüller, 2012; Schieber and Chandel, 2014; Nisa et al., 2019). Therefore, ROS-scavenging mechanisms, including the expression of antioxidant genes such as *ascorbate peroxidase* (*APX*), *catalase* (*CAT*), *glutathione peroxidase* (*GPX*), and *superoxide dismutase* (*SOD*), are crucial for defending against oxidative stress. ROS levels in plants are extensively increased by abiotic stresses, such as drought, cold, and salinity, which promote the accumulation of ROS in plants (Moran et al., 1994; Agnihotri and Seth, 2017; Huang et al., 2019; Wu et al., 2019; Mariyam et al., 2023a, b; Choudhary et al., 2024; Kumar et al., 2024a). This suggests that ROS accumulation is involved in plant response and tolerance to abiotic stresses, and experimental results using knock-out mutations and the overexpression of antioxidant genes have demonstrated this. Zhang et al. (2013) showed that *OsAPX2* mutant and *OsAPX2*-overexpressing transgenic rice exhibited elevated and reduced ROS levels, respectively, compared with wild-type rice under abiotic stress conditions (Zhang et al., 2013). Correspondingly, *OsAPX2* mutant rice exhibited hypersensitivity to drought stress, whereas *OsAPX2*-overexpressing rice displayed enhanced tolerance. Similarly, plants that overexpress *CAT* enzymes, such as *PtCAT2*, *OsCATa*, and *OsCATc*, displayed enhanced tolerance to drought stress (Joo et al., 2014; Xu et al., 2023). These results indicate the crucial role of antioxidant gene expression in managing ROS accumulation and enhancing plant tolerance under abiotic stress conditions. This is further supported by previous research, which revealed that the expression of antioxidant genes and ROS accumulation are regulated by jasmonic acid (JA), a key phytohormone that mediates plant defense against both abiotic and biotic stresses (Abdelgawad et al., 2014; Brenya et al., 2020; Kim et al., 2021; Sheteiwy et al., 2021).

Despite growing evidence that PGPRs, including *Bacillus velezensis*, improve plant tolerance to abiotic stress, our understanding of the underlying mechanisms remains limited. In this study, we show that the *B. velezensis* strain GH1-13 enhances drought tolerance by promoting the expression of ROS-scavenging genes and suppressing ROS accumulation. Furthermore, our findings suggest that the activation of the JA response is deeply involved in this process.

## Materials and methods

### Plant materials and growth

Two varieties of japonica rice, *Oryza sativa* cv. *Shindongjin* and cv. *Saechungmu*, were used in this study. Rice seeds were sterilized with 2.5% sodium hypochlorite (v/v) for 1 h and then washed five times with sterile distilled water. The sterilized seeds were germinated on half-strength Murashige and Skoog (^1^/_2_ MS) solid medium for 3 days. They were then transplanted and grown in soil under normal growth chamber conditions (16 h/8 h = light/dark, 32°C).

### Microbe materials, growth, and treatment

*B. velezensis* GH1-13 has been previously described (Kim et al., 2017, 2019; Lee et al., 2023), and for this study, GH1-13 was obtained from the Center for Industrialization of Agricultural and Livestock Microorganisms. For the GH1-13 treatment of rice, GH1-13 was cultivated in a tryptic soy broth (glucose 0.5%, soybean flour 0.8%, NaCl 0.15%, K_2_HPO_4_ 0.25%, Na_2_CO_3_ 0.05%, and MgSO_4_·7H_2_O 0.1%) at 30°C for 36 h in a shaking incubator and collected by centrifugation. The rice was treated with GH1-13 at a final concentration of 1 × 10^7^ CFU/mL.

### Drought and JA treatment

For drought treatment, the plants were exposed to dehydration conditions for 3 days. Following this 3-day drought treatment, the plants were re-watered and cultivated under normal growth conditions (16 h/8 h = light/dark, 32°C). The survival rate was calculated by dividing the number of plants that survived by the total number of plants that were tested. For JA treatment, a methyl jasmonate (MeJA) solution [100 μM MeJA in double-distilled water (ddH_2_O) and 0.1% Tween-20 (v/v)] was applied to the rice by spraying, and the plants were incubated under normal growth conditions for 12 h.

### Measurement of chlorophyll contents

The chlorophyll content was measured as previously described by Wellburn (1994) with slight modifications (Wellburn, 1994). To extract the chlorophyll, fresh leaves (0.3 g) were collected from the indicated plants and incubated with 10 mL of 80% acetone (v/v) in the dark for 24 h. After incubation, the samples were centrifuged at 12,000 ×*g* for 5 min, and the supernatant was diluted 10-fold with 80% acetone. The chlorophyll contents were measured spectrophotometrically at wavelengths of 663 and 646 nm (Molecular Devices VersaMax Microplate Reader) and calculated according to the method described by Wellburn (1994).

### Visualization of ROS levels by DAB and NBT staining

To visualize the ROS levels in rice, the histochemical detection of H_2_O_2_ and O_2_^·-^ was performed using diaminobenzidine (DAB) and nitroblue tetrazolium (NBT) staining. For DAB and NBT staining, leaves collected from the indicated plants were incubated in DAB staining solution [1 mg/mL 10 mM Na_2_HPO_4_ (pH 3.0)] and NBT staining solution [0.1% (w/v) nitroblue tetrazolium in 10 mM sodium azide and 50 mM potassium phosphate buffer (pH 6.4)] at room temperature overnight in the dark. After incubation, the leaves were incubated in a bleaching solution (ethanol:glycerol:acetic acid at a ratio of 3:1:1, v/v/v) to bleach out the chlorophyll. To quantify NBT staining intensity, the formazan contents were measured in the NBT-stained samples. The NBT-stained samples (0.3 g) were ground with liquid nitrogen and then the formazans were extracted from the ground samples using an extraction solution (2 M potassium hydroxide:DMSO at a ratio of 1:1.6, v/v). The formazan contents were measured with a spectrophotometer at a wavelength of 630 nm (Molecular Devices VersaMax Microplate Reader) and calculated as previously described by Grellet Bournonville and Díaz-Ricci (2011).

### Total RNA extraction and quantitative RT-PCR analysis

To analyze the relative transcript levels, total RNA was extracted from the indicated plants using the RNeasy Plant Mini Kit (Qiagen) according to the manufacturer’s instructions. The first complementary DNA (cDNA) strand was synthesized using 1 µg of total RNA, oligo dT primers, and GoScript Reverse Transcriptase (Promega). For quantitative RT-PCR (RT-qPCR), a master mix was prepared using the AccuPower GreenStar qPCR Master Mix (Bioneer). The PCR reaction and fluorescence detection were performed with a CFX Connect Real-Time System (Bio-Rad). Three technical replicates of the RT-qPCR were performed using three biological replicates. The RT-qPCR conditions included an initial denaturation at 95°C for 5 min, followed by 45 cycles of denaturation at 95°C for 10 s, annealing at 58°C for 10 s, and extension at 72°C for 10 s. *OsACTIN1* was used as the internal control. Primer sequence information is available in **Supplementary Table 1**.

### Embedding, sectioning, and toluidine blue staining

To observe the internal anatomy of the rice roots, Technovit embedding and physical sectioning were performed as previously described by Seo et al. (2021) with slight modifications. Rice root samples were collected from the indicated plants and fixed in 4% paraformaldehyde solution (w/v) for 2 h, and then washed five times with ddH_2_O. The fixed samples were dehydrated in an ethanol series [25%, 50%, 75%, and 100% (v/v) in ddH_2_O] for 2 h at each step. The dehydrated samples were incubated in a series of Technovit 7100 cold-polymerizing resin solutions [25%, 50%, 75%, and 100% (v/v) in ethyl alcohol] for 2 h at each step. Samples were further incubated in a 100% Technovit 7100 solution for 1 day and solidified in a 15:1 (v/v) mixture of Technovit 7100 and hardener solution II at room temperature in a mold for 1 day. Sections (3 μm) were obtained from solidified samples using an RM 2145 microtome (Leica). The sections were stained with 0.05% toluidine blue solution (w/v, pH 4.5) for 1 min and observed with a light microscope (DM-2500, Leica).

### Statistical analysis

Quantification data for the RT-PCR and chlorophyll contents are the averages of three biological replicates with three technical replicates each. The survival rate results are presented as the mean values of at least three biological replications. Statistical analysis was performed using Microsoft Excel 365 MSO (version 2406, Build 16.0.1726.20078). Statistical differences between the samples and their respective controls were determined using a two-tailed Student’s *t-*test with *p <* 0.01.

## Results

### *Bacillus velezensis* GH1-13 improves drought tolerance in rice

**Figure 1:**
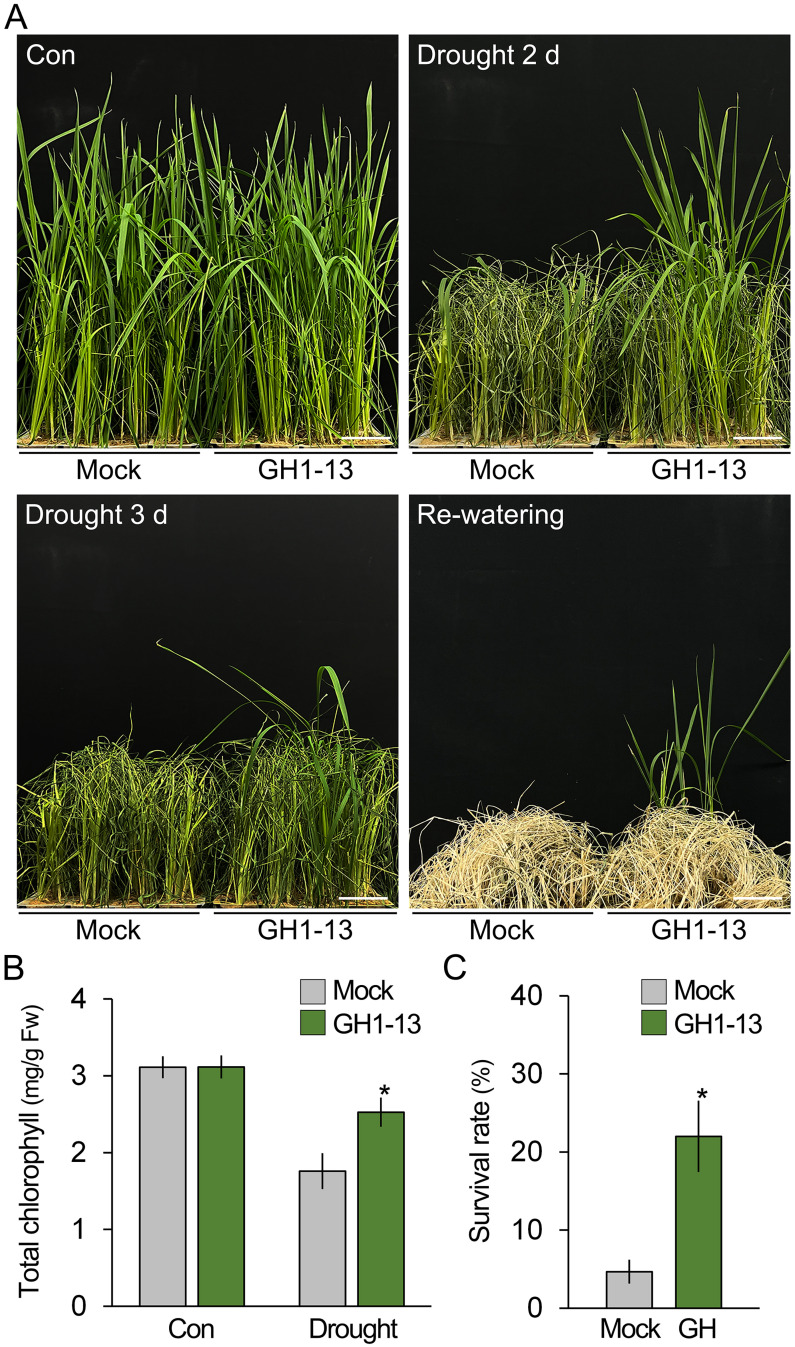
*Bacillus velezensis* GH1-13 improves drought stress tolerance in rice. **(A)** Phenotypic analysis of drought stress tolerance in 8-week-old rice (cv. *Shindongjin*) treated with and without *B. velezensis* GH1-13. Rice was exposed to drought stress for 3 days and subsequently re-watered and cultivated under normal growth conditions for 10 days. **(B)** Effects of GH1-13 treatment on the chlorophyll content of rice treated and untreated with drought stress for 2 days. Mock and GH1-13 indicate *B. velezensis* GH1-13-untreated and -treated rice for 7 days, respectively. **(C)** Survival rates of the re-watered rice plants (*n* > 250). The survival rate was calculated by dividing the number of surviving plants by the total number of plants tested. Error bars indicate SD. Asterisks indicate statistically significant differences between the corresponding samples and their control (*p* < 0.01, *t*-test). Scale bars = 5 cm.

To investigate the effect of *B. velezensis* GH1-13 on drought stress tolerance in rice, 8-week-old rice (cv. *Shindongjin*) plants were treated and untreated with GH1-13 and exposed to drought conditions, and their phenotypical and physiological changes were monitored over time (**Figure 1**). After 2 days of drought stress, both the GH1-13-treated and -untreated plants exhibited symptoms of drought stress, including chlorosis, leaf rolling, and wilting. However, the visual symptoms in the GH1-13-treated rice were milder compared to those of the untreated rice, and the quantification results of total chlorophyll contents supported this observation (**Table 1**). Unlike the GH1-13-untreated rice, in which drought stress reduced the total chlorophyll contents by 43.5%, the GH1-13-treated rice exhibited an 18.9% total chlorophyll content decrease under drought stress conditions. This suggests that GH1-13 treatment enhances rice tolerance to drought stress, and the survival rates of the re-watered rice supported this. When these plants were re-watered and grown for 10 days, the rice treated with GH1-13 exhibited an approximately 4.7-fold increase in survival rate compared to that of untreated rice. The effect of GH1-13 on rice drought tolerance was also evident in another rice cultivar, *Saechungmu* (**Supplementary Figure 1**; **Table 1**). Similar to *Shindongjin* rice, GH1-13 treatment significantly improved drought stress tolerance in *Saechungmu* rice, which highlights the role of GH1-13 in enhancing rice tolerance to drought stress.

**Table 1:** The effect of GH1-13 on chlorophyll content and survival rate in rice.

Rice cultivar	Conditions		Total chlorophyll (mg/g Fw)	Survival rate (%)
*Shindongjin*	Con	Mock	3.11 ± 0.14	–
		GH1-13	3.12 ± 0.17	–
	Drought	Mock	1.76 ± 0.23	4.67 ± 1.53
		GH1-13	2.53 ± 0.17*	22.00 ± 4.58*
*Saechungmu*	Con	Mock	3.07 ± 0.14	–
		GH1-13	3.13 ± 0.15	–
	Drought	Mock	1.65 ± 0.19	9.67 ± 3.05
		GH1-13	2.37 ± 0.18*	36.67 ± 5.13*

Asterisks indicate statistically significant differences between the corresponding samples and their control (*p* < 0.01, *t*-test).

### *Bacillus velezensis* GH1-13 reduces ROS accumulation

**Figure 2:**
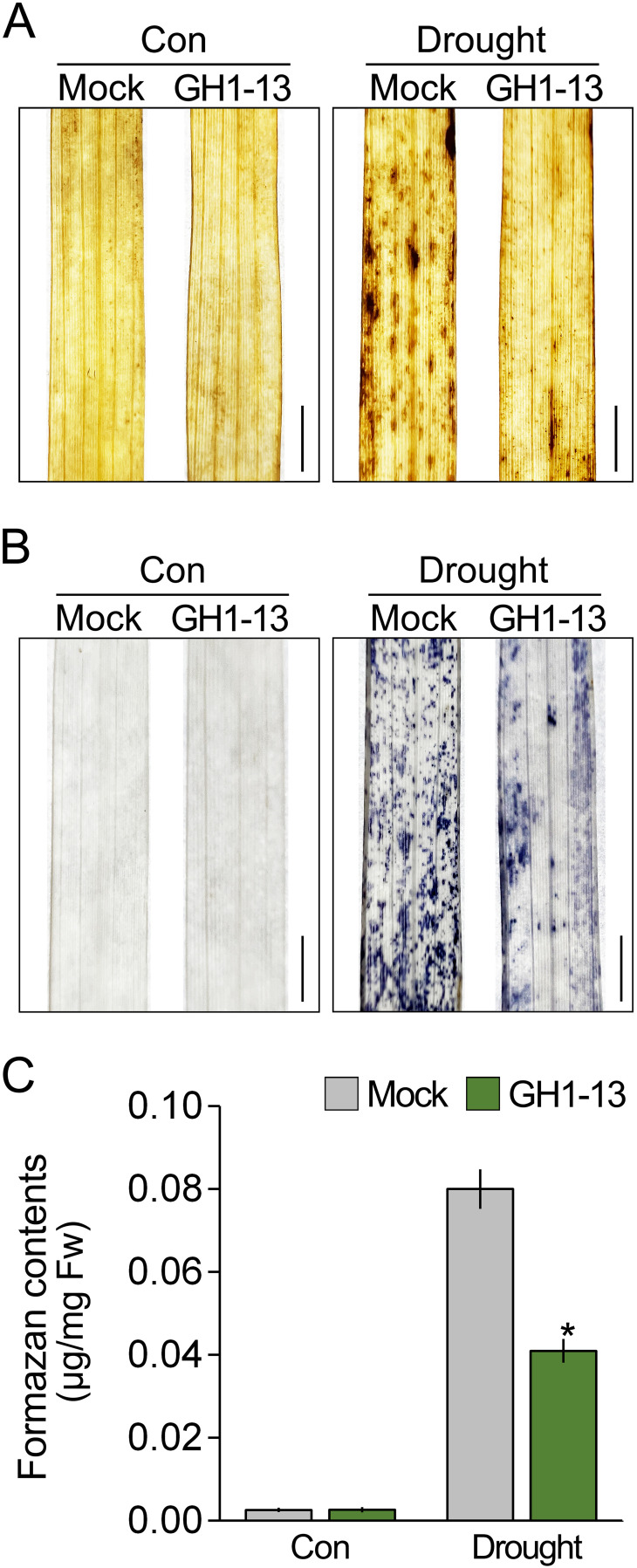
*B. velezensis* GH1-13 reduces drought-induced ROS accumulation. DAB **(A)** and NBT staining analysis **(B)** visualized the effect of GH1-13 treatment on ROS accumulation under drought stress. Rice was exposed to drought stress for 2 days. Mock and GH1-13 indicate *B. velezensis* GH1-13-untreated and -treated rice for 7 days, respectively. **(C)** Quantification of formazan contents to quantify the NBT staining intensity in these plants. Error bars indicate SD. An asterisk indicates statistically significant differences between the corresponding samples and their control (*p* < 0.01, *t*-test). Scale bars = 0.5 cm.

The regulation of ROS accumulation is closely linked to plant tolerance to abiotic stress (Moran et al., 1994; Mittler, 2002; Sharma et al., 2012; Huang et al., 2019; Wu et al., 2019; Kim et al., 2021; Sun et al., 2021). Since GH1-13 improves drought stress tolerance in rice, we expected that GH1-13 treatment would affect ROS accumulation under drought stress conditions. To investigate this, we visualized the accumulated ROS using DAB staining (**Figure 2A**). Under normal growth conditions, there was no difference in DAB staining intensity between the untreated and treated rice with GH1-13. However, under drought conditions, the GH1-13-treated rice exhibited reduced intensity compared to the untreated rice, which suggests that the GH1-13 treatment diminishes drought-induced ROS accumulation. To explore this further, the ROS levels in these plants were visualized and quantified using NBT staining (**Figures 2B, C**). Consistent with the DAB staining results, the intensity of NBT staining in the GH1-13-treated rice was markedly lower than that in the untreated rice under drought stress conditions. Quantification of NBT staining intensity by measuring the formazan content revealed that the NBT staining intensity in the GH1-13-treated rice was 48.88% lower than that in the GH1-13-untreated rice, thus indicating that GH1-13 treatment reduces ROS accumulation under drought stress conditions.

### GH1-13 promotes expressions of ROS-scavenging and JA-responsive genes

**Figure 3:**
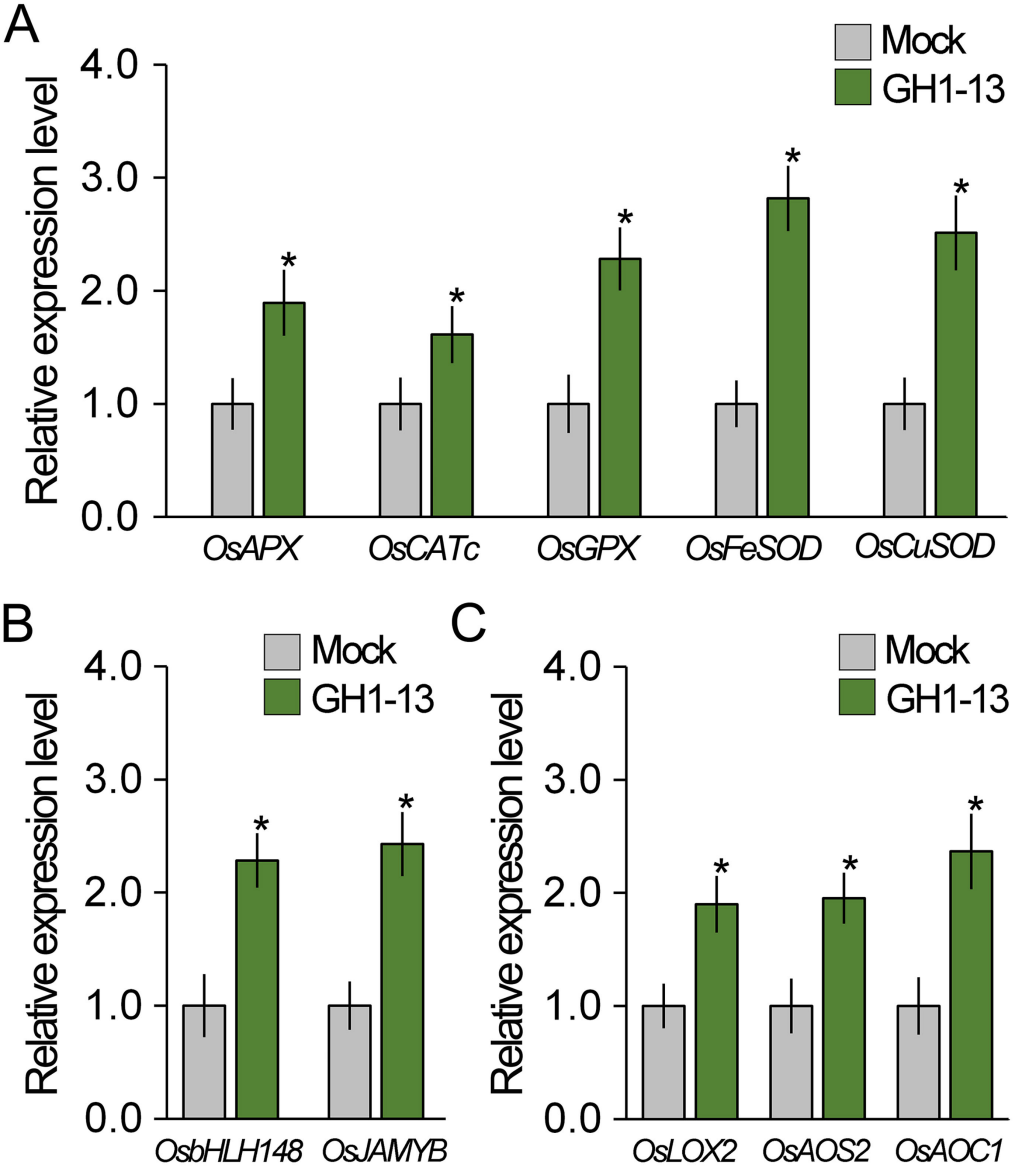
GH1-13 treatment activates the expression of ROS-scavenging and JA-responsive genes. **(A)** Quantitative RT-PCR results showing the expression of ROS-scavenging genes in *B. velezensis* GH1-13-treated and -untreated 8-week-old rice. **(B, C)** Expression levels of JA signaling **(B)** and biosynthesis genes **(C)** in *B. velezensis* GH1-13-treated and -untreated rice. Mock and GH1-13 indicate *B. velezensis* GH1-13-untreated and -treated rice for 7 days, respectively. *OsACTIN1* was used as the reference gene to normalize the RT-qPCR results. Error bars indicate SD. Asterisks indicates statistically significant differences between the corresponding samples and their control (*p* < 0.01, *t*-test).

Since *B. velezensis* GH1-13 reduces ROS accumulation and enhances drought stress tolerance, we hypothesized that GH1-13 treatment influences the expression of antioxidant genes such as rice *ascorbate peroxidase* (*OsAPX*), *catalase* (*OsCAT*), *glutathione peroxidase* (*OsGPX*), and *superoxide dismutase* (*OsSOD*). To test this hypothesis, we quantitatively analyzed the transcript levels of ROS-scavenging genes in rice treated with and without GH1-13 using RT-qPCR (**Figure 3A**). Our finding revealed that the GH1-13-treated rice exhibited an increased expression of ROS-scavenging genes compared to the untreated rice, which explains the reduced ROS levels in the rice treated with *B. velezensis* GH1-13.

Since JA is deeply involved in plant–microbe communication and regulates the expression of antioxidant genes (Van der Ent et al., 2009; Carvalhais et al., 2013; Fan et al., 2016; Farooq et al., 2016; Liu et al., 2017; Sheteiwy et al., 2021; Zhu et al., 2022), it was hypothesized that GH1-13 possibly affects JA response. To address this, the JA response was examined in GH1-13-treated and -untreated rice by analyzing the transcript levels of JA-responsive genes responsible for JA signaling (O*sbHLH148* and *OsJAMYB*) and for JA biosynthesis (*OsLOX2*, *OsAOS2*, and *OsAOC1*) (Seo et al., 2011; Yang et al., 2020; Qiu et al., 2022) (**Figures 3B, C**). The expression level of JA-responsive genes in the GH1-13-treated rice was significantly higher than that of the untreated rice, which suggests that *B. velezensis* GH1-13 activates the JA response in rice.

### Jasmonic acid treatment enhances drought tolerance in rice

**Figure 4:**
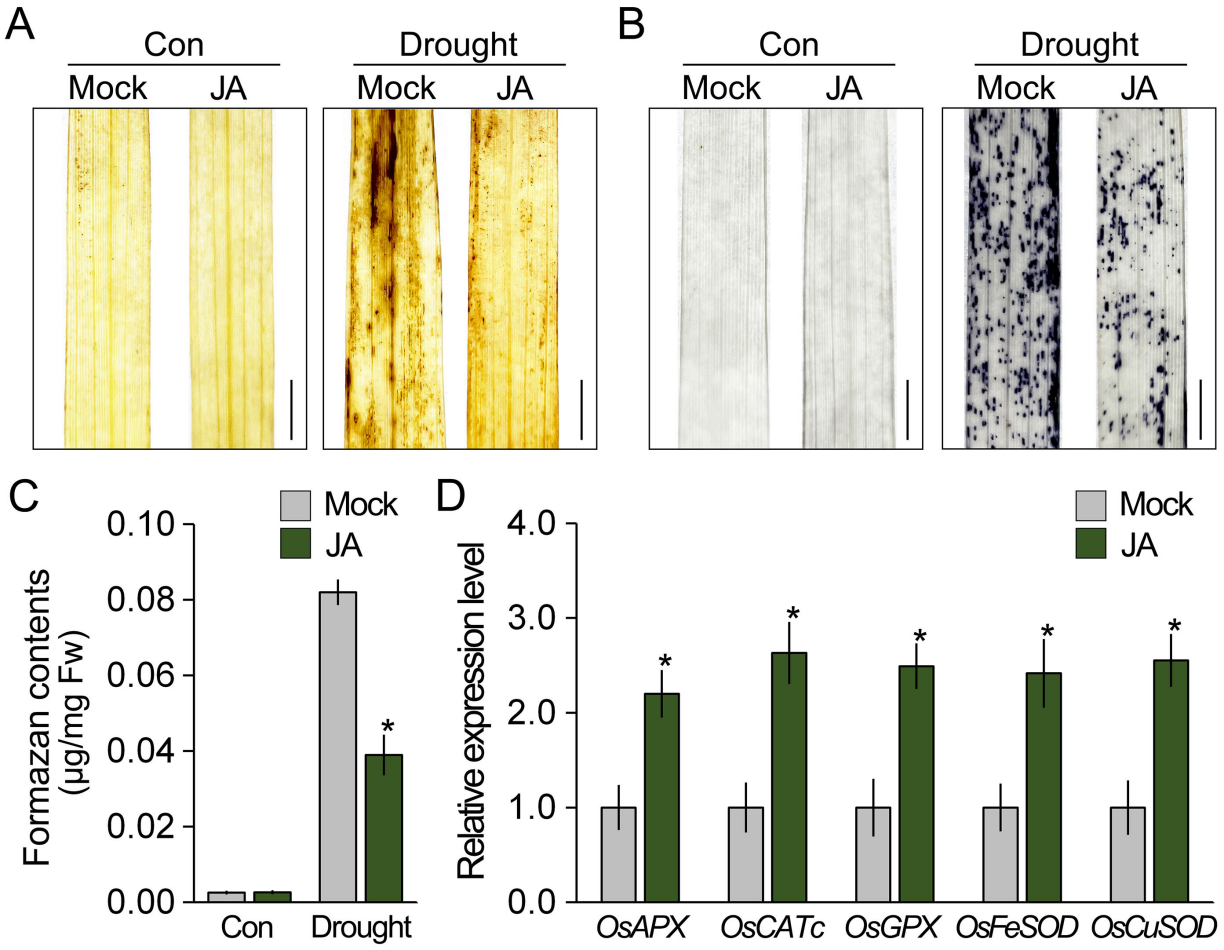
JA reduces the accumulation of ROS in rice. The effect of JA on ROS accumulation was visualized using DAB **(A)** and NBT staining **(B)**. Visualization of ROS accumulation. Rice treated and untreated with JA for 12 h was exposed to drought stress for 2 days. **(C)** Measurement of formazan contents to quantify the NBT staining intensity in these plants. **(D)** Changes in the expression of ROS-scavenging genes by JA treatment. Total RNA was extracted from 7-week-old rice treated and untreated with JA for 12 h, and *OsACTIN1* was used as the reference gene to normalize the RT-qPCR results. Error bars indicate SD. Asterisks indicates statistically significant differences between the corresponding samples and their control (*p* < 0.01, *t*-test). Scale bars = 0.5 cm.

Since JA response is deeply involved in abiotic stress tolerance and ROS homeostasis in plants, it was expected that JA affects ROS accumulation in rice under drought stress conditions. To test this, we treated 7-week-old rice with JA for 12 h and then analyzed the ROS levels under both normal and drought conditions using DAB and NBT staining (**Figures 4A-C**). Although drought stress increased the intensity of the DAB and NBT staining in both the JA-treated and -untreated rice, the intensity in the JA-untreated rice was significantly higher than that in the JA-treated rice under drought conditions. These findings indicated that the ROS levels in JA-treated rice are lower than those in the untreated rice under drought stress conditions, which suggests that JA diminished the accumulation of drought-induced ROS. In addition, we found that transcript levels of ROS-scavenging genes in the JA-treated rice were higher than those in the JA-untreated rice (**Figure 4D**). These results indicate that JA activates the expression of ROS-scavenging genes and suggest that JA reduces ROS accumulation by activating the expression of ROS-scavenging genes.

**Figure 5:**
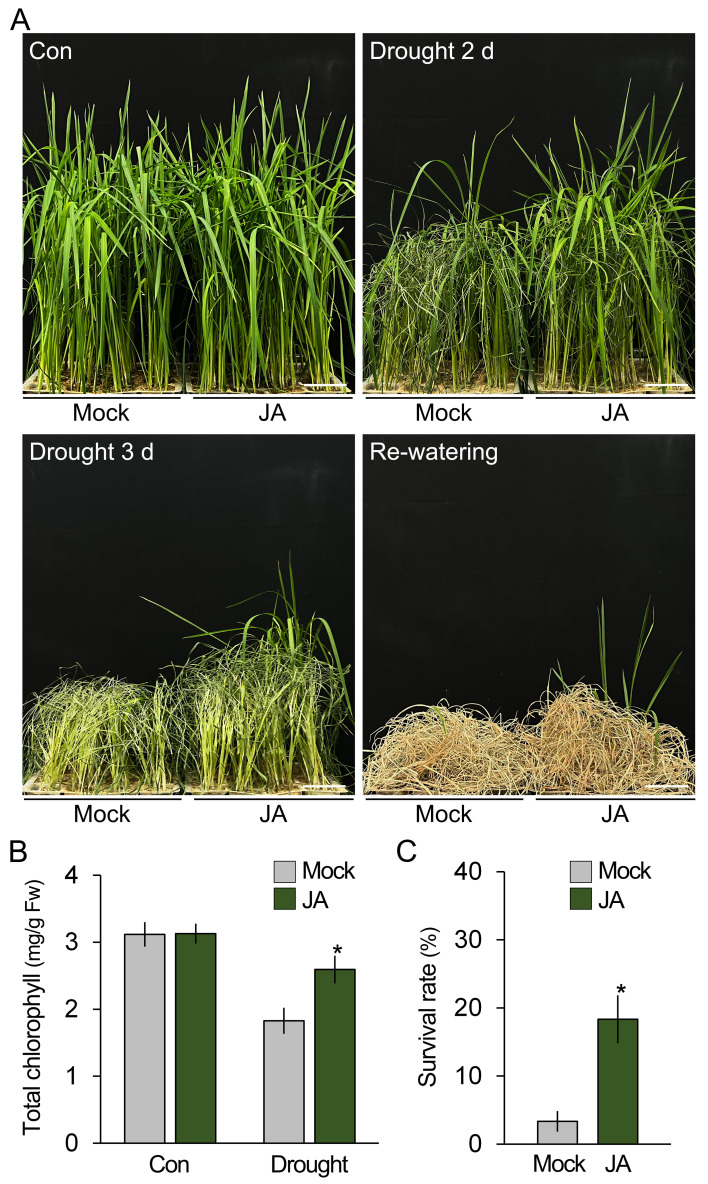
Drought tolerance is enhanced by JA treatment. **(A)** Phenotypic analysis of drought stress tolerance in 7-week-old rice (cv. *Shindongjin*) treated and untreated with JA. Rice, both treated and untreated with JA, was exposed to drought stress for 3 days and then cultivated under re-watered conditions for 10 days. **(B)** Effects of exogenous JA treatment on the chlorophyll content of rice treated and untreated with drought stress for 2 days. Mock and JA indicate exogenous JA-untreated and -treated rice, respectively. **(C)** Survival rates of the re-watered rice (*n* > 250). The survival rate was calculated by dividing the number of surviving plants by the total number of plants tested. Error bars indicate SD. Asterisks indicate statistically significant differences between the corresponding samples and their control (*p* < 0.01, *t*-test). Scale bars = 5 cm.

Because JA, like *B. velezensis* GH1-13, promoted the expression of antioxidant genes and reduced the accumulation of ROS, it was hypothesized that JA treatment would be sufficient to enhance the drought stress tolerance of rice. To test this, JA-treated and -untreated 7-week-old rice plants were exposed to drought stress, and their phenotypical and physiological changes were analyzed (**Figure 5**). Although both sets of plants exhibited obvious visual symptoms of drought stress, such as leaf rolling and wilting, these symptoms were less severe in the JA-treated rice compared to the untreated rice. In addition, these observations were supported by the results showing that the chlorophyll content and survival rates were higher in the JA-treated rice compared to the untreated rice. Together with the finding that *B. velezensis* GH1-13 activates the JA response, these results suggest that *B. velezensis* GH1-13 enhances drought stress tolerance in rice by reducing ROS accumulation, and the activation of the JA response is deeply involved in this process.

### Analysis of root growth in rice treated with GH1-13

**Figure 6:**
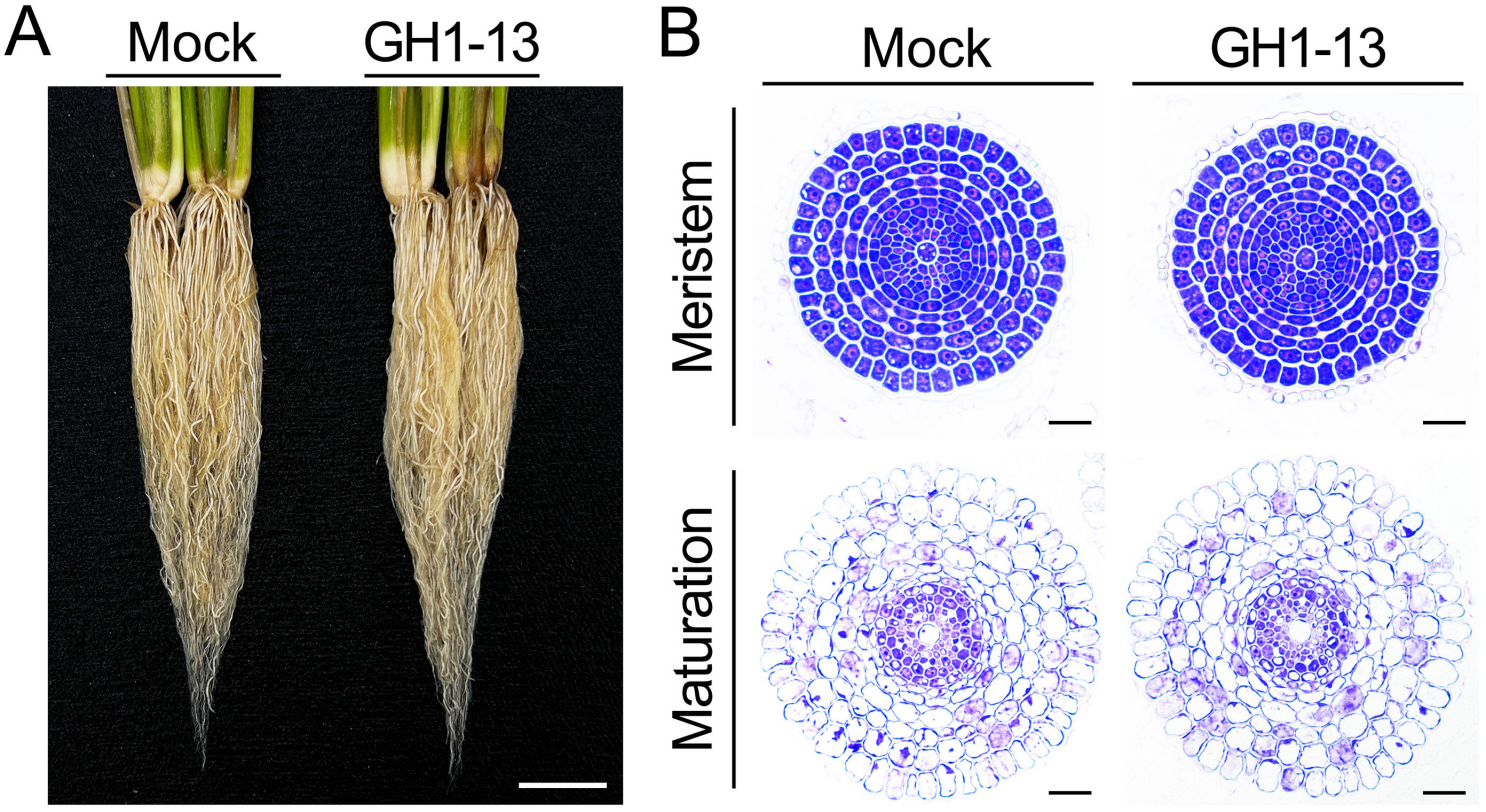
Effect of GH1-13 on rice root growth. **(A)** Root development of *B. velezensis* GH1-13-treated and -untreated 8-week-old rice. **(B)** Transverse section images of the roots. Mock and GH1-13 indicate *B. velezensis* GH1-13-untreated and -treated rice for 7 days, respectively. Scale bars = 2 cm in **(A)** and 50 μm in **(B)**.

Roots are essential organs that interact with soil microbes, and drought stress tolerance is influenced by root growth (Ekanayake et al., 1985; Seo et al., 2020; Grover et al., 2021; Khoso et al., 2023). To determine whether root development contributes to the enhanced tolerance by *B. velezensis* GH1-13 treatment, we analyzed the root morphology of rice plants treated with and without GH1-13. When comparing the phenotypes of 8-week-old roots grown under GH1-13-treated and -untreated conditions, no significant differences were observed in root growth, including root length (**Figure 6A**). In addition, experimental results obtained from 2-week-old roots were consistent with those from 8-week-old roots (**Supplementary Figure 2**). To further investigate this, we analyzed the internal anatomy of the roots through physical sectioning and microscopic observation (**Figure 6B**). No significant differences in root development, including root radial patterning, were observed between the treated and -untreated roots in the root meristem and maturation zones. This suggests that GH1-13 treatment improves drought stress tolerance in rice by regulating physiological processes, such as inhibiting ROS accumulation, rather than influencing root development.

## Discussion

In this study, we showed that *B. velezensis* GH1-13 enhances drought stress tolerance in rice by activating the expression of antioxidant genes and suppressing ROS accumulation. Previous studies have revealed that ROS levels are extensively increased by stresses. For example, tomatoes treated with drought conditions and *Botrytis cinerea* exhibited approximately 2.5- and 3-fold increased ROS levels, respectively, compared to the untreated control plants (Pietrowska et al., 2015; Gowtham et al., 2020). This suggests that plant responses and tolerance to stress are closely linked to the accumulation of ROS, and studies investigating ROS-scavenging genes, such as *APXs*, *CATs*, and *SODs*, further support this (Zhang et al., 2013; Xu et al., 2023). The expression level and the activity of ROS-scavenging genes were affected by abiotic and biotic stresses, such as drought and *Pseudomonas syringae*, and knock-out mutations of antioxidant genes, such as *APXs*, *CATs*, and *SODs*, resulted in the increased accumulation of ROS and reduced tolerance to abiotic stresses (Moran et al., 1994; Mittler et al., 1999; Bueso et al., 2007; Dvořák et al., 2021). In contrast, the overexpression of these antioxidant genes led to a decrease in ROS accumulation and enhanced tolerance in plants, such as Arabidopsis and rice (Joo et al., 2014; Li et al., 2015, 2017; Xu et al., 2023). Furthermore, the overexpression of drought-responsive *IbMYB116* and *VaNAC17* genes decreased ROS levels by promoting the transcriptional expression of ROS scavenging genes and enhanced drought stress tolerance (Zhou et al., 2019; Su et al., 2020). These results indicate that ROS accumulation plays a crucial role in plant tolerance to abiotic stresses and support our finding that *B. velezensis* GH1-13 enhances drought stress tolerance in rice by activating the expression of ROS-scavenging genes and suppressing the accumulation of ROS.

JA is a phytohormone that plays a key role in mediating the plant’s defense against abiotic stresses, as demonstrated by previous studies involving the endogenous modulation of the JA response (Seo et al., 2011; Grebner et al., 2013; Wu et al., 2015; Fu et al., 2017; Xing et al., 2020; Zhang et al., 2023; Kumar et al., 2024b). A study by Seo et al. (2011) showed that the overexpression of *OsbHLH148*, a key transcription factor in JA signaling, enhanced drought tolerance by activating the JA response in rice (Seo et al., 2011). A study by Fu et al. (2017) revealed that a knock-out mutation of *OsJAZ1*, a negative regulator of JA signaling, improved drought tolerance in rice, whereas the overexpression of *OsJAZ1* reduced drought tolerance compared to wild-type rice (Fu et al., 2017). Consistently, modulation of the JA response through knock-out mutations and overexpression of JA biosynthetic genes, such as *LOXs* and *AOSs*, affects plant tolerance to drought stress. For example, a knock-out mutation of *AtLOX6* decreased JA levels, which led to hypersensitivity to drought stress in *Arabidopsis thaliana* (Grebner et al., 2013). In contrast, the overexpression of *CmLOX10* and *BoAOS* increased JA levels and improved drought stress tolerance (Wu et al., 2015; Xing et al., 2020). These findings indicate that activation of the JA response is closely associated with plant tolerance to drought stress.

Furthermore, the crucial role of JA in plant tolerance to drought stress is supported by its impact on the expression of antioxidant genes and the suppression of ROS accumulation. In various crops, such as Indian mustard, wheat, maize, tobacco, millet, and soybeans, JA treatment upregulated the expression of ROS-scavenging genes, such as *APXs*, *CATs*, and SODs, while reducing the accumulation of ROS (Örvar et al., 1997; Abdelgawad et al., 2014; Anjum et al., 2016; Agnihotri and Seth, 2020; Awan et al., 2021; Sheteiwy et al., 2021). Similarly, the activation of the JA response through the overexpression of *SlCOI1* or *MdLOX3* reduced ROS accumulation by promoting the expression of ROS-scavenging genes (Chen et al., 2024; Kadam and Barvkar, 2024). Conversely, suppression of the JA response through *SlCOI1* knockdown reduced the JA response, which resulted in a decreased expression of ROS-scavenging genes and increased ROS accumulation (Kadam and Barvkar, 2024). In this study, we showed that GH1-13-treated rice exhibited an increased expression of JA-responsive and ROS-scavenging genes compared to the untreated control plants. Taken together with the tight correlation of JA, ROS accumulation, and drought tolerance, our findings suggest that *B. velezensis* GH1-13 enhances rice drought tolerance by activating the expression of antioxidant genes and reducing ROS accumulation, and activation of the JA response is involved in this process. Accumulating evidence, including the findings from this study, suggests that PGPRs could serve as a key strategy to enhance crop drought tolerance. However, challenges remain, such as optimizing field application conditions and accurately predicting PGPR responses within field environments. Further studies on the mechanisms underlying PGPR-induced tolerance and the practical aspects of their field application will be crucial for unlocking the full potential of PGPRs as effective microbial fertilizers.

## Conclusion

This study highlights the role of *B. velezensis* GH1-13 in enhancing drought stress tolerance in rice. Molecular characterization, including ROS accumulation and antioxidant gene expression assessments, indicates that GH1-13 reduces ROS accumulation under drought stress by increasing the expression of antioxidant genes. Furthermore, this finding is supported by evidence that GH1-13 treatment enhances the JA response, which activates the expression of antioxidant genes. Collectively, this study concludes that the ROS-scavenging process mediated by antioxidant genes is a key mechanism that underlies the improved drought stress tolerance in rice conferred by *B. velezensis* GH1-13.

## Data Availability

The original contributions presented in the study are included in the article/**Supplementary Material**. Further inquiries can be directed to the corresponding author.
