# Supplementary material for: Bacillus velezensis GH1-13 enhances drought tolerance in rice by reducing the accumulation of reactive oxygen species
Source: Front Plant Sci. 2024 Sep 25;15:1432494. doi: 10.3389/fpls.2024.1432494 (PMC11465243; doi:10.3389/fpls.2024.1432494)
Supplement: Supplementary file 1 [file DataSheet1.pdf]

Supplementary Material

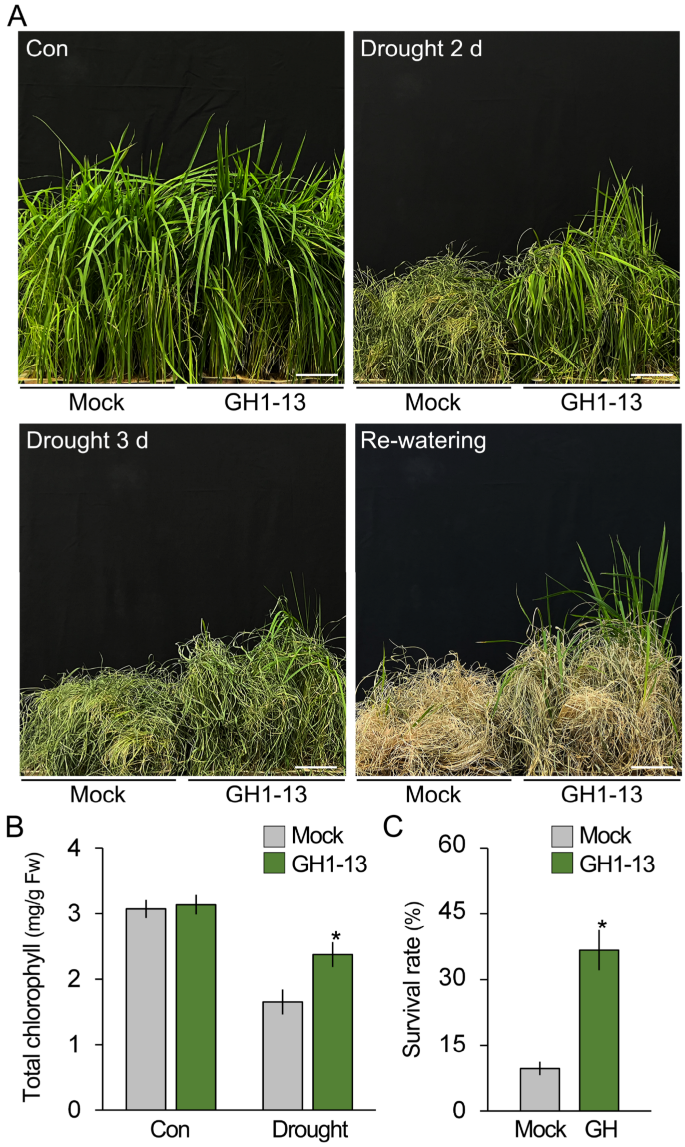

Supplementary Figure 1. Enhanced drought tolerance by GH1-13 in another rice cultivar. **(A)** Phenotypic analysis of drought stress tolerance in 7-week-old rice (cv. Saechungmu) treated with and without *Bacillus velezensis* GH1-13. Rice exposed to drought stress for the indicated time (2 and 3 days) was subsequently re-watered and cultivated under normal growth conditions for 10 days. **(B)** Effects of GH1-13 treatment on chlorophyll contents in rice treated and untreated with drought stress for 2 days. Mock and GH1-13 indicate *Bacillus velezensis* GH1-13-untreated and -treated rice for1 week, respectively. **(C)** Quantification of survival rates of the re-watered rice (n>250). Survival rate was calculated by dividing the number of plants that survived by the total number of plants tested. Error bars indicate SD. Asterisks indicate statistically significant differences between the corresponding samples and their control (p value < 0.01, t-test). Scale bars = 5 cm.

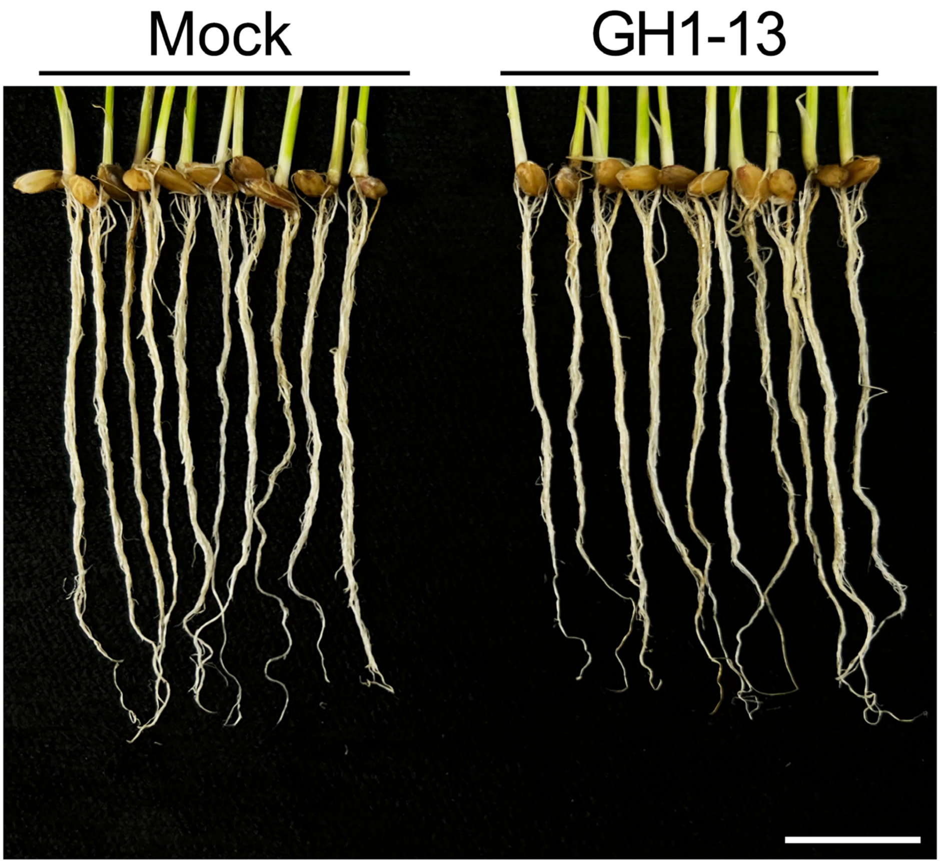

Supplementary Figure 2. Root growth in rice treated with GH1-13. Root growth in GH1-13-untreated (Mock) and -treated (GH1-13) rice. One-week-old rice was treated and untreated with GH1-13, and cultivated under normal growth conditions for 1 week. Scale bar = 2 cm.

Supplementary Table 1. Primers used in this study.

Name	Sequence
OsAPX RT 5 For	CTTTGAAGACTACAAGGAGG
OsAPX RT 3 Rev	GGGCAATGTACTAGCAGTAG
OsCAT RT 5 For	GAGGTCGACTACTTCCCATC
OsCAT RT 3 Rev	GCTTGAAGTTGTTCTCCTTG
OsGPX RT 5 For	TGGGAGCTCTTAAACCTAAA
OsGPX RT 3 Rev	CCTGTGAAGTACATGTCACC
OsFeSOD RT 5 For	TGGCACAGAATTCTGTCATT
OsFeSOD RT 3 Rev	AATGGATGCGAAAATATCGC
OsCuSOD RT 5 For	CCATTTGGGAGTCAAGCTAA
OsCuSOD RT 3 Rev	TCCTCACTGTATCCATGCTT
OsbHLH148 RT 5 For	TTGTGAAGTGGAAAAGGCAG
OsbHLH148 RT 3 Rev	TTTGCACATGAGATGTCTGT
OsJAMYB RT 5 For	TTGGTCTACAGAGATTCAGC
OsJAMYB RT 3 Rev	GACTCCATAGAGTTTCGGAC
OsLOX2 RT 5 For	GCTTACTACCGTAACCACTG
OsLOX2 RT 3 Rev	CATTCGCGTATGGGTAGTC
OsAOS2 RT 5 For	CTAGCGTTGACAACAAGCAG
OsAOS2 RT 3 Rev	GGAGGTTGAAGCTTTGGTG
OsAOC1 RT 5 For	TGACCTACGAGGAGTCCTAC
OsAOC1 RT 3 Rev	CCTTGAGGTAGAAGGTGTAG
OsIAA3 RT 5 For	GTGCTGAGAACCAGAAAGAT
OsIAA3 RT 3 Rev	CCTTTCATGATTCTGAGCCT
OsIAA9 RT 5 For	CAATGATGATCATGGCGATG
OsIAA9 RT 3 Rev	GTTTCACAGATCGTGCAAAA
OsRR6 RT 5 For	ACGTCAACATGATCATCACC
OsRR6 RT 3 Rev	TTCTCCGACGACATGATCAC
OsRR9 RT 5 For	ACTACCAGCAGGAACAAAAC
OsRR9 RT 3 Rev	CTTTCTCTTGTGGCTGTTGC
OsACTIN1 RT 5 For	CACTATGTTCCCTGGCATTG
OsACTIN1 RT 3 Rev	CTGTACTTCCTTTCAGGAGG
